# Constructing Safety at Home: Parents’ Experiences of the First Hospital-to-Home Transition in Childhood Cancer

**DOI:** 10.3390/nursrep16100348

**Published:** 2026-09-25

**Authors:** Sofia Silva, Isabel Quelhas, Esperança Gago

**Affiliations:** 1 Faculty of Health Sciences and Nursing, Universidade Católica Portuguesa, 4169-005 Porto, Portugal; iquelhas@ucp.pt; 2 Department of Nursing, Santa Maria Health School, 4049-024 Porto, Portugal; 3 School of Nursing, University of Minho, 4710-057 Braga, Portugal; epereira@ese.uminho.pt

**Keywords:** childhood cancer, family-centred care, hospital-to-home transition, paediatric nursing, parents, safety

## Abstract

**Background/Objectives**: The first hospital discharge during childhood cancer treatment marks a transition from continuous professional monitoring to greater parental responsibility at home. This study aimed to explore parents’ experiences of this hospital-to-home transition and how they sought to establish safety while assuming responsibility for care. **Methods**: This qualitative study presents a focused analysis of data from a broader grounded theory study conducted according to Corbin and Strauss at a single specialised paediatric oncology centre in Portugal. The broader study included 16 parents recruited through theoretical sampling; the present analysis included 14 participants whose interviews provided substantive accounts of the discharge transition relevant to this analytical category. Data were generated through open interviews and analysed using constant comparison and progressive category development. **Results**: The first hospital discharge was characterised by relief and renewed family connection alongside uncertainty, perceived vulnerability, and fear of clinical deterioration. Parents sought to construct safety at home through vigilance, environmental adaptation, preventive practices, organisation of complex care, and mobilisation of professional and informal support. Four interrelated subcategories were identified: transitioning beyond professional monitoring, establishing safety at home, assuming responsibility for complex care, and protecting the child while sustaining family life. **Conclusions**: Safety following discharge was not simply restored by returning home but progressively constructed through parental action, learning, and adaptation. Nursing support should extend beyond technical discharge instruction to include progressive preparation, assessment of parental readiness, development of caregiving competence, accessible nursing guidance, multidisciplinary support, and attention to family conditions influencing sustainable care at home. This study was not registered.

## 1. Introduction

Childhood cancer affects not only the child but the entire family system, requiring sustained adaptation to treatment demands, clinical uncertainty, disruptions to everyday life, and changes in family functioning [1,2,3]. Parents commonly assume a central caregiving role throughout the illness trajectory, while also managing the emotional impact of the disease, and the wider demands imposed on family life [4,5]. These demands may affect parental well-being and require substantial adjustments to family responsibilities and employment, with consequences that may persist beyond active treatment [6,7,8].

Within the disease trajectory, the transition from hospital to home represents a clinically significant change in both the context of care and the distribution of responsibility between healthcare professionals and families. Continuity of care across this transition depends on effective preparation, communication, and coordination, while the absence of formal mechanisms linking hospital and community care may compromise care continuity after discharge [9]. In paediatric oncology this transition is particularly relevant as parents must incorporate treatment-related care into family everyday life while simultaneously assuming responsibilities that were previously undertaken or closely supervised by healthcare professionals.

Preparation for discharge is, therefore, an important component of paediatric oncology care. Research on families of newly diagnosed children has demonstrated the importance of structured, nurse-led discharge education and support, addressing symptom management and parental coping [10]. However, the challenges of this transition extend beyond the initial acquisition of knowledge prior to discharge, as once at home, parents must interpret clinical guidance, recognise changes in the child’s condition, organise treatment-related tasks, and judge when professional advice should be sought or when the child requires clinical reassessment, while also managing the demands of everyday family life.

Recent research directly examining the hospital-to-home transition in childhood cancer reinforces the complexity of this process. Perceived discharge safety has been described as depending not only on the provision of appropriate education, medications, and supplies, but also on healthcare professionals’ assessment of caregivers’ medical and psychosocial readiness to return home [11].

Qualitative evidence among mothers of children with leukaemia further indicates that the transition from hospital to home can be characterised by fear, doubt, perceived vulnerability, and uncertainty as responsibility for care increasingly shifts to the family [12]. These findings suggest that readiness for discharge cannot be reduced to the completion of clinical teaching but is instead dependent on parents’ developing capacity to manage uncertainty and responsibility in the absence of healthcare professionals.

The assumption of caregiving responsibilities at home occurs within a family system already affected by the demands of childhood cancer. Treatment often requires a redistribution of responsibilities, adjustments to employment, and changes to family routines and relationships [2,3,8]. These effects extend beyond parents and the child undergoing treatment. Siblings may experience reduced parental availability, shifts in family roles and routines, and challenges across home, school, and social environments [13,14,15]. The transition home should therefore be understood not only as a transfer of clinical responsibility but also as a complex process through which families balance the child’s care and protection with the continuity of family life.

This perspective is consistent with family-centred care approaches in paediatric nursing, which recognise the family as an active partner in care and emphasise information sharing, participation, collaboration, and respect for family circumstances and capabilities [16,17,18]. Parental participation, however, should not be equated with an unsupported transfer of responsibility. Supporting the transition home requires nursing attention to parents’ developing caregiving competence, confidence in decision-making, access to timely nursing and multidisciplinary guidance, and the family conditions required to sustain care over time [19].

Although previous research has identified important aspects of discharge preparation, continuity of care, caregiver readiness, fear, uncertainty, and the practical demands associated with returning home [9,10,11,12], less attention has been paid to how parents actively establish a sense of safety once continuous professional monitoring is no longer immediately available. In particular, the ways in which parents respond to clinical vulnerability and uncertainty by reorganising caregiving practices, the home environment, family interactions, and available support have not been fully conceptualised as an ongoing process. Understanding how these actions converge during the transition home may provide a more comprehensive account of how parents assume and sustain responsibility for care following discharge.

This study therefore aimed to explore parents’ experiences of the hospital-to-home transition following their child’s first hospital discharge during cancer treatment and to examine how they sought to establish safety while assuming responsibility for care at home.

## 2. Materials and Methods

This section describes the methodological procedures of the broader grounded theory study and the focused analytical process underpinning the present analysis.

### 2.1. Study Design

This qualitative study presents a focused analysis of data generated within a broader grounded theory study exploring families’ experiences across the childhood cancer trajectory. The broader study followed the grounded theory approach described by Corbin and Strauss [20] and aimed to develop a substantive nursing theory explaining the processes experienced by families throughout childhood cancer.

Within the broader analysis, the child’s first hospital discharge and subsequent transition home emerged as a major analytical category. For the purposes of this focused analysis, the category “first hospital discharge” referred to the child’s first discharge home following diagnosis and an initial period of inpatient treatment. In one analytically specific case involving haematopoietic stem cell transplantation (HSCT), the transition analysed concerned a subsequent discharge in the child’s illness trajectory—namely, the first discharge home following transplantation. This case was included because the intensity and specificity of post-transplant caregiving and infection-prevention demands were analytically relevant to the category. The present article examines this category in depth, focusing on parents’ experiences of assuming responsibility for care and establishing safety at home. Its analytical relevance reflected the convergence of parental actions, conditions, and consequences around the transfer of care from hospital to home, supporting its examination as a distinct transition within the broader illness trajectory. Within this focused analysis, safety is understood as an experiential construct reflected in parents’ perceived capacity to protect their child, manage care-related risks, and respond to uncertainty at home, rather than as an objectively measured clinical outcome.

The study is reported in accordance with the Consolidated Criteria for Reporting Qualitative Research (COREQ) [21] (Supplementary File S1).

### 2.2. Setting and Participants

The broader study was conducted in a specialised paediatric oncology unit within a national referral centre in Portugal. The unit provides inpatient and outpatient care, including haematopoietic stem cell transplantation. The institution is not identified to protect institutional and participant confidentiality.

Participants were mothers and fathers of children diagnosed with cancer who were receiving active treatment, had completed curative treatment, or had died. Parents were eligible if they were willing to participate and able to provide reflective accounts of their experiences across the childhood cancer trajectory.

The broader grounded theory study included 16 parents—10 mothers and 6 fathers—selected through theoretical sampling. Sampling occurred concurrently with data collection and analysis and sought variation in the child’s clinical trajectory, family circumstances, access to support, and experiences of treatment completion or bereavement.

One father declined participation, and two mothers who had initially expressed willingness to participate could not be interviewed due to logistical constraints.

The present focused analysis included 14 participants whose interviews contained substantive accounts of the discharge transition relevant to this analytical category. Two participants from the broader study did not contribute because their joint interview did not contain material on this transition that could contribute meaningfully to the analysis. For the purposes of the focused analysis, a substantive account was understood as one containing experiential and contextual detail concerning the discharge home, parental responsibilities, perceived risks, caregiving actions, or consequences of the transition. The 14 participants comprised 9 mothers and 5 fathers and represented families across a range of childhood cancer diagnoses and clinical trajectories, with varying levels of care complexity. Participant, interview, and child characteristics are summarised in Table 1.

Participants included in the present analysis were assigned alphanumeric codes indicating parental role (M for mother and F for father), followed by a sequential number. These codes are used to attribute the quotations presented in the findings.

### 2.3. Data Collection

Data collection for the broader study took place between May 2022 and June 2025. Data were generated through open interviews with parents and reflective and analytical memos produced by the lead researcher.

Potential participants were initially contacted by telephone and informed about the study purpose and procedures. After agreeing to participate, they selected the interview time and location. Interviews were conducted in participants’ homes or workplaces, private rooms within the healthcare institution, or public locations chosen by participants. Only the participant or participants and the lead researcher were present during interviews.

Parents could participate individually or, when both parents agreed, as a couple. Fourteen interviews were conducted with 16 participants. Two couples were interviewed together, three couples participated in separate interviews, and the remaining interviews involved one parent. Interviews lasted an average of approximately 1 h and 45 min. The interval between the analysed discharge and the interview ranged from approximately 8 months to 7 years, reflecting participants’ different positions in the childhood cancer trajectory.

All interviews were conducted in Portuguese by the lead researcher, audio-recorded with permission, and transcribed verbatim by the researcher without automated transcription software. Transcripts were checked against the recordings to ensure accuracy.

No repeat interviews were conducted. Transcripts were not returned to participants, and the findings were not submitted for formal participant validation. Field notes were written immediately after interviews and incorporated into the reflective and analytical memos.

An open interview guide was used to support data generation and was progressively refined throughout concurrent data collection and analysis, in keeping with the iterative logic of grounded theory (Supplementary File S2). The guide was not formally pilot tested. Interviews initially followed an open and exploratory format, becoming progressively more focused on emerging concepts and relationships as the analysis advanced. Because the first hospital discharge emerged as analytically relevant during the broader grounded theory analysis, it was not explored through an identical set of questions with every participant. All interviews addressed the broader childhood cancer trajectory, while later interviews increasingly explored the first discharge and transition home as their analytical relevance became clearer. The depth and emphasis of individual accounts therefore reflected both participants’ experiences and the evolving analytical focus of the study. In joint interviews, individual contributions were identified in the transcripts and distinguished analytically where possible, while areas of agreement, elaboration, and divergence between partners were also considered. The individual parent account was treated as the primary unit of analysis, while interaction between partners in the joint interview was also considered analytically.

Reflective and analytical memos documented contextual observations, provisional interpretations, methodological decisions, and questions requiring further exploration.

### 2.4. Data Analysis

Data collection and analysis occurred concurrently, following the grounded theory procedures described by Corbin and Strauss [20]. The broader analysis involved constant comparison and a progressive movement from initial coding to category development and integration. NVivo (Release 14.23.2, Build 46; Lumivero, Denver, CO, USA) was used solely to support the organisation and management of data, codes, and categories developed manually by the lead researcher.

As the first hospital discharge and transition home gained analytical relevance within the broader study, subsequent interviews increasingly explored the conditions, actions, interactions, and consequences associated with this transition. Later data therefore contributed to refining its properties and dimensions and to examining variation in parental preparedness, care complexity, perceived vulnerability, and available support.

With the first hospital discharge and transition home established as a major analytical category within the broader study, all related interview segments, codes, and analytical memos were retrieved and examined in greater depth for the present focused analysis. Incidents were compared within and across the accounts of the 14 contributing participants to identify similarities, differences, conditions, parental actions and interactions, and perceived consequences. Codes were reviewed and refined, and their conceptual relationships were examined to clarify how parents responded to reduced professional monitoring, interpreted risk, reorganised the home, developed caregiving competence, and sought to sustain family life.

This focused analysis resulted in the development of the overarching category constructing safety at home, which integrated four interrelated subcategories: transitioning beyond professional monitoring, establishing safety at home, assuming responsibility for complex care, and protecting the child while sustaining family life. This analytical structure reflected the progressive conceptual integration of incident-level codes into higher-order subcategories and the overarching category.

Adequate category development was assessed through the recurrence and variation in relevant incidents across accounts, the increasing stability of relationships among the four subcategories, and the point at which additional data no longer substantially modified the conceptual properties of the overarching category within the available dataset. These criteria informed the assessment of theoretical sufficiency within the focused analysis, rather than treating saturation as a numerical threshold.

Some participants’ discharge experiences occurred during a period in which COVID-19-related public health measures remained in place. Because infection-prevention practices were also integral to paediatric oncology care, particularly during neutropenia and after HSCT, specific behaviours were attributed to the pandemic only when participants explicitly linked them to COVID-19.

Coding and conceptual development were conducted by the lead researcher, with emerging codes, subcategories, the overarching category, and analytical relationships regularly discussed with the supervisory team.

Meleis’ Transition Theory, Orem’s supportive–educative nursing system, uncertainty in illness, and family-systems perspectives were not used to guide coding or category development. These theoretical perspectives were introduced only after category development to support interpretation of the findings in Section 4. Discussion.

Generative artificial intelligence (ChatGPT, GPT-5.6 Sol; OpenAI) was used solely to assist in the preparation of the conceptual figure and was not used for data collection, coding, analysis, or interpretation. The final figure was reviewed and verified by the authors.

### 2.5. Rigour and Reflexivity

Rigour was supported through prolonged engagement with the data, repeated reading of transcripts, constant comparison, analytical memo writing, and the grounding of interpretations in participants’ accounts. An audit trail documented sampling decisions, coding development, analytical refinements, and the relationships established among concepts. Consideration of alternative interpretations through regular analytical discussion with the supervisory team supported the credibility and dependability of the analysis.

The lead researcher was a female nurse and doctoral researcher, a specialist in child and paediatric health nursing, with a Master’s degree in Nursing, professional experience in paediatric oncology, and training in qualitative research. She had previously known some participants in a professional capacity. This prior familiarity was disclosed to participants and was recognised as a source of contextual knowledge and theoretical sensitivity, while also representing a potential influence requiring sustained reflexive attention. At the time of recruitment and interview, however, she was not directly responsible for their clinical care and did not provide nursing care to any of the participating families after their interviews. Participation was presented as entirely voluntary and independent of clinical care, and participants were explicitly informed that declining participation would have no consequences for their care or relationship with the healthcare team. Reflexivity was supported through memo writing and regular analytical discussion with the supervisory team. Detailed description of the setting, participants, data collection procedures, and analytical process was provided to support readers’ assessment of transferability. These procedures were consistent with the criteria of credibility, transferability, dependability, and confirmability proposed by Lincoln and Guba [22].

### 2.6. Ethical Considerations

Ethical approval was granted by the institutional Health Ethics Committee on 18 November 2021 (Ref. CES.262/021). An amendment permitting the inclusion of bereaved parents was approved on 19 November 2024 (Ref. CES.223/024).

Participation was voluntary, and written informed consent was obtained before data collection. Participants received written and verbal information about the study purpose, procedures, possible emotional demands, confidentiality protections, and their right to withdraw at any time without consequence.

Given the sensitivity of discussing a child’s cancer trajectory, interviews could be paused, postponed, or terminated at the participant’s request. Identifying information was removed from transcripts and from the presentation of findings. Illustrative quotations were translated from Portuguese into English by the lead researcher. Translations were checked against the original Portuguese transcripts to preserve semantic meaning, tone, and contextual nuance, with wording reviewed with the other researchers where clarification was required.

The institution is not identified because the study was conducted in a highly specialised paediatric oncology setting in which disclosure could increase the possibility of indirect participant identification. Original data were accessible only to the lead researcher and stored in password-protected files in accordance with the approved protocol.

## 3. Results

The analysis showed that the first hospital discharge marked a transition from professionally monitored care to greater parental responsibility at home. Returning home was associated with relief and renewed family connection, but also with uncertainty, fear of clinical deterioration, and concern about managing care without the immediate presence of healthcare professionals. Parents sought to establish safety through heightened vigilance, reorganisation of the home environment, acquisition of care-related knowledge, regulation of social contact, and mobilisation of professional and informal support. Four interrelated subcategories were identified, representing complementary dimensions through which parents progressively constructed safety at home: transitioning beyond professional monitoring, establishing safety at home, assuming responsibility for complex care, and protecting the child while sustaining family life (Figure 1).

### 3.1. Transitioning Beyond Professional Monitoring

The first hospital discharge represented a meaningful milestone for parents, signalling that the child’s clinical condition allowed for a return home and offering the possibility of renewed family life. Nevertheless, relief and hope coexisted with apprehension. Leaving the hospital meant moving away from an environment characterised by continuous professional observation, immediate clinical support, and established care routines. At home, parents anticipated having to recognise changes in the child’s condition and make decisions without the immediate presence of healthcare professionals.

This transition was particularly unsettling when the discharge occurred sooner than parents had expected or when there was little time between being informed of the decision and leaving the hospital. One mother described her reaction when she unexpectedly learned that her child would be discharged that day:

“At that moment, it was such a shock for me: ‘What? I’m going home today? Oh my God!’ I completely lost it.”

(M2)

Although returning home was generally desired, some parents did not initially experience it as an unequivocal sign of safety. Instead, the hospital had become the place where risks could be rapidly identified and managed, whereas the home was perceived as a less controlled environment in which potential threats were more difficult to anticipate. Information concerning neutropenia and infection risk heightened this perception of vulnerability and sometimes led parents to interpret ordinary aspects of the home environment as possible sources of danger:

“When [the mother] told me, ‘Her defences are very low; we have to be very careful,’ we almost panicked. I remember thinking that perhaps it would be better not even to open the window, so that no kind of virus could come in.”

(F2)

The absence of continuous professional monitoring also intensified fears of clinical deterioration. Parents questioned whether they would recognise warning signs, respond appropriately, or reach the hospital in time. For some, this uncertainty prompted heightened observation of the child and the acquisition of monitoring devices as a means of compensating for the perceived loss of professional surveillance:

“What if something happens to her and I’m not at the hospital? I think that is a common fear. We only realised with time that she was not about to die at any moment, but it was difficult. I bought a pulse oximeter.”

(M3)

Parents’ experiences varied according to how prepared they felt, previous caregiving experience, clarity of information received, and ability to contact the healthcare team after discharge. Continued access to nursing guidance, within the wider multidisciplinary team, allowed parents to clarify doubts and validate decisions, partially extending the sense of clinical support beyond the hospital. Conversely, limited preparation or uncertainty about when and how to seek help intensified anticipatory anxiety and hypervigilance.

Transitioning beyond professional monitoring was therefore not experienced as a simple movement from hospital to home. It involved emotional and cognitive adjustment, as parents had to reconcile the reassurance associated with discharge with an increased awareness of the child’s vulnerability. Over time, repeated experience and continued professional accessibility contributed to greater confidence. In the initial period, however, parents remained suspended between the relief of returning home and the fear of becoming primarily responsible for identifying and responding to clinical risk.

### 3.2. Establishing Safety at Home

Once at home, parents began to view ordinary household objects, spaces, and routines through a clinical lens. Because potential sources of infection were often perceived as difficult to identify, establishing safety involved reorganising the domestic environment and introducing preventive practices intended to make risk more visible and manageable.

For some families, this process involved extensive physical reorganisation. Curtains, rugs, and other objects perceived as possible sources of contamination were removed or covered, while cleaning and disinfection practices became more intensive:

“That house was turned upside down. I removed all the curtains, the rugs, everything. I covered the sofas with sheets.”

(F2)

Household spaces and objects were also repurposed to accommodate infection-prevention practices. One mother described how an everyday item in the kitchen became entirely dedicated to cleaning materials:

“We had a trolley in the kitchen with cookbooks. It was emptied and filled from top to bottom with disinfecting products. The house started to look like a hospital.”

(M5)

Through these adaptations, families translated uncertainty and largely invisible risk into concrete actions. The protective logic associated with the hospital was partially transferred into the domestic setting, giving the home a hybrid character: it remained the centre of family life while also becoming organised according to clinical recommendations and preventive routines.

The extent of this transformation varied. Some parents introduced limited adjustments that were gradually incorporated into daily life, whereas others reorganised the home extensively and maintained highly demanding routines. These differences reflected the child’s clinical condition, the guidance received, parents’ perception of risk and previous knowledge, and the material characteristics and resources of the household. Environmental adaptation could increase reassurance while simultaneously adding to domestic workload and emotional strain.

For some families, perceived infection risk led to particularly intensive cleaning practices and the acquisition of equipment specifically intended to reduce environmental contamination:

“We gave no chance to any germ that might exist in the house. Our house was immaculate. We even bought a steam cleaner to sterilise the floor.”

(F4)

Food preparation also became a significant source of anxiety. In one analytically specific account, the transition concerned a subsequent discharge in the child’s illness trajectory—the first discharge home following HSCT. In this context, food-related infection-prevention requirements intensified parental fear of causing harm:

“The nurse who discharged us told me, ‘You have to be very careful with food because a bacterium in the food could be fatal.’ My God, could I kill my daughter? I washed everything, washed it again, and scalded it. I was so afraid of cooking for her.”

(M6)

In this post-transplant account, repeated checking, sterilisation, and strict adherence to protective guidance became central to everyday care. These practices reflected the parent’s efforts to respond responsibly to the child’s vulnerability, but could also become highly restrictive and emotionally exhausting.

Establishing safety at home was therefore an evolving process in which reassurance was pursued through environmental reorganisation and preventive routines. Greater perceived clinical vulnerability was associated with more extensive domestic adaptation, intensifying both the protective structure surrounding the child and the practical and emotional demands placed on parents.

### 3.3. Assuming Responsibility for Complex Care

Beyond adapting the home environment, parents became responsible for coordinating and delivering care that had previously been supported by hospital routines and healthcare professionals. Medication administration, food preparation, hygiene, symptom observation, and daily schedule organisation now had to be integrated into family life. For many parents, this transfer of responsibility was experienced as abrupt and demanding.

One mother described the contrast between the hospital, where care was embedded within an established system, and the home, where the family had to recreate that structure independently:

“Now I have to extend all of this to home? How am I going to do it? At the hospital, there is already a whole system in place, and then at home we have to organise that entire system ourselves.”

(M3)

Assuming responsibility for care required rapid learning and continuous adjustment. Parents had to understand instructions, remember multiple tasks, establish routines, and coordinate care alongside ordinary household and family responsibilities. The complexity of these demands was not limited to technically difficult procedures; it also arose from the accumulation of tasks and the perceived consequences of error.

For some parents, this responsibility was closely connected to their sense of themselves as mothers or fathers. Difficulties in managing food, hygiene, or medication were therefore not experienced merely as practical challenges, but as possible failures in their parental role:

“At the hospital, food and cleaning were not my responsibility. Here at home, I had to know how to manage all of that. I became very worried; I was very afraid of failing as a mother.”

(M6)

The effort required to remain attentive to every aspect of care also contributed to physical and emotional overload:

“When you leave the hospital, you have to be attentive to everything. You have to cook, manage the medication—it is overwhelming.”

(M4)

Parents responded by developing increasingly structured ways of managing care. They organised medication schedules, established routines, recorded care-related information, and divided tasks when another caregiver was available. These strategies contributed to transforming a complex and initially unfamiliar set of responsibilities into a more manageable pattern of daily care. One mother described recording detailed information because the volume and pace of events made it difficult to rely on memory:

“I wrote everything down. There came a point when I could no longer remember anything because there was so much pressure and so much happening, almost every hour.”

(M2)

The experience varied according to the complexity of the child’s care, parents’ previous knowledge and health literacy, the clarity of discharge preparation, and access to professional guidance when doubts arose. For some parents, care responsibilities became progressively integrated into everyday life. For others, the combination of continuous vigilance, practical demands, and fear of making a harmful mistake remained highly burdensome.

Assuming responsibility for complex care was therefore a process of gradual competence-building rather than an immediate achievement. Parents moved from uncertainty and fear of failure towards greater organisation and confidence through learning, repeated practice, and the progressive integration of care into daily routines. Even as care became more familiar, the responsibility attached to each decision remained emotionally significant.

### 3.4. Protecting the Child While Sustaining Family Life

Returning home allowed the child to reunite with siblings, grandparents, and other significant family members. However, these reunions occurred within a family environment already altered by the child’s clinical vulnerability. Parents had to balance the desire to restore closeness and normal family life with the perceived need to regulate contact and minimise infection risk.

Interactions that would ordinarily have been spontaneous became mediated by protective rules. Physical proximity, affection, and shared spaces were negotiated through measures such as mask use, distancing, and restrictions on contact. One mother described the unfamiliarity of being together again as a family while continuing to follow protective guidance:

“We had been there for so long that, when we came home and the four of us were together, it felt strange. It was our house, you know? And then there were the rules—the brother hugged her, but he was wearing a mask.”

(M5)

Contact with extended family was also carefully controlled. Parents sometimes restricted relatives to brief or physically distanced encounters, despite the importance of these relationships:

“My parents came to the window, and so did my parents-in-law. We hardly allowed them to come near her.”

(M8)

These arrangements were intended to protect the child but also transformed expressions of affection and belonging. Family reunification was therefore not a return to previous patterns of interaction. It involved regulated intimacy, in which closeness remained valued but was continually negotiated against perceived clinical risk.

Protecting the child also depended on the family’s capacity to reorganise everyday responsibilities. The demands of care, household management, employment, and attention to other children could rarely be sustained by one parent alone. When available, partners, grandparents, other relatives, friends, colleagues, and paid support helped distribute practical tasks and created space for parents to rest or attend to other family needs:

“Fortunately, we had someone who helped with the cleaning, and my mother cooked every day, so that was one less meal for me to worry about. My father took care of many things related to our work. Her father managed part of the medication and came home a little earlier to play with [the child], so that I could have some space for myself.”

(M5)

Support was often most useful when it was practical, predictable, and compatible with the protective measures surrounding the child. Friends and relatives adapted the way they provided assistance, offering help without entering the home or coming into close contact with the child:

“I did not do the shopping. We lived on the ground floor. I told my colleagues what I needed, and they left the bags on the balcony. I collected them and waved goodbye from a distance.”

(M5)

However, access to support was uneven. Some parents relied almost exclusively on their partner, without extended family available to provide meals, household assistance, or respite:

“I do not have support from anyone else. My support is my husband; it is just the three of us. I do not have anyone preparing a meal for me when I come home.”

(M3)

Differences in financial resources, family structure, and the availability of informal or institutional support influenced how responsibilities were distributed. When support was limited, care and household demands became concentrated within the parental unit, reducing opportunities for rest and self-care. Some parents described giving up personal activities and sources of comfort in order to prioritise the child and maintain family functioning.

The tension between protection and connection was particularly evident when protective measures restricted contact with extended family and friends. Strict control of visits and prolonged isolation were intended to reduce infection exposure but could gradually weaken social contact. One mother described how visitors initially complied with protective measures but became less present over time:

“People (…) always wore a mask. But over time, they no longer wanted to come.”

(M7)

Protective boundaries could therefore support parents’ efforts to minimise perceived risk while simultaneously narrowing the family’s social world. As restrictions continued, parents faced the risk of increasing isolation precisely when sustained practical and emotional support was most needed.

Protecting the child while sustaining family life required families to continually negotiate competing needs rather than resolve them completely. Parents regulated closeness, redistributed responsibilities, and adapted available support in an effort to preserve family relationships while seeking to minimise perceived risk to the child. Effective and flexible support helped sustain family functioning, whereas its absence or gradual withdrawal intensified isolation and concentrated the practical and emotional burden within the immediate family.

## 4. Discussion

The findings indicate that the first hospital discharge during childhood cancer treatment is shaped by a central tension: returning home enables renewed family life while simultaneously transferring greater responsibility, uncertainty, and perceived risk to parents. This study showed that managing this transition involves the progressive construction of safety, the development of caregiving competence, and the reorganisation of family life around the child’s clinical vulnerability. These findings extend existing accounts of discharge readiness and caregiving burden by showing how these dimensions are interconnected within a broader parental process of constructing safety at home.

### 4.1. Constructing Safety Beyond Professional Monitoring

The first hospital discharge marked a critical transition in the childhood cancer trajectory, involving not only a change in care setting but also a reconfiguration of parental roles, responsibilities, and perceived competence. This interpretation is consistent with Meleis’ Transition Theory, which conceptualises transition as a multidimensional process shaped by changes in identity, behaviour, relationships, and expectations [23,24]. The coexistence of relief and apprehension reflects the ambiguity of this transition: returning home represented progress and renewed family life but also meant leaving the continuous professional monitoring that had come to signify safety.

The findings indicate that safety was not automatically restored through discharge. Rather, it was actively constructed by parents through vigilance, preventive practices, environmental adaptation, and continued access to nursing and multidisciplinary guidance. In this context, safety was not experienced as the absence of risk, but as parents’ growing capacity to recognise, organise, and respond to risk within everyday family life. This extends existing literature on family adaptation in childhood cancer, which describes adjustment as a dynamic process influenced by clinical demands, parental resources, care-related competence, and contextual conditions [25,26,27,28]. During the early transition home, parental actions were directed towards making perceived risk more observable and manageable.

This process can be understood through the concept of uncertainty in illness. When threats could not be fully predicted or controlled, parents sought to preserve their capacity to act by translating uncertain risks into observable and manageable practices. Such responses are consistent with forms of situated control through which families manage uncertainty without eliminating it [29,30]. Similar strategies have been described as mechanisms through which families reduce disorganisation and regain a sense of direction during childhood cancer treatment [31,32,33].

A distinctive finding was the transformation of the home into a hybrid care environment. Although home remained the centre of family life, its spaces, objects, and routines were progressively reorganised according to infection-prevention requirements and perceived clinical risk. This tension between preserving continuity and incorporating illness-related demands is consistent with systemic perspectives on family adaptation, which emphasise the reciprocal reorganisation of family functioning in response to disease [34,35,36].

Although these adaptations could increase reassurance, they also intensified domestic workload and, in some cases, contributed to restrictive and emotionally demanding routines. In the analytically specific post-HSCT account, these adaptations were particularly intensive, reflecting the heightened caregiving and infection-prevention demands associated with this transition. Preventive guidance should therefore be clinically precise, proportionate, and tailored to both the child’s level of risk and the family’s living conditions. Without such calibration, risk-focused information may inadvertently amplify fear or encourage practices beyond those required for safety.

The findings also reinforce the need for discharge preparation to extend beyond information provision immediately before leaving hospital. Family-centred care and partnership approaches emphasise shared information, negotiated responsibilities, and progressive parental participation rather than unsupported transfer of care [16,18,37]. Preparation should therefore include anticipatory guidance, clear criteria for recognising deterioration and seeking help, and an accessible route of communication with the healthcare team during the early period at home [19].

### 4.2. Developing Parental Competence for Complex Care

Assuming responsibility for complex care required parents to move beyond knowing what should be done towards being able to interpret, organise, and perform care safely in everyday situations. Competence was not acquired at a single point before discharge; rather, it developed progressively through practice, routine-building, and the adjustment of knowledge to the child’s condition and the realities of family life. This finding challenges approaches that reduce parental preparation to the transmission of clinical information.

Family-centred care and partnership models recognise parents as central participants in paediatric care while also emphasising that responsibilities should be negotiated according to families’ preferences, resources, and developing capabilities [16,18,37]. In the present study, assuming care was accompanied by fear of error and concern about being unable to respond adequately. Parental participation should therefore not be equated with immediate readiness or competence. Instead, it should be understood as a negotiated process in which responsibility increases as parents acquire the knowledge, practical skills, and confidence required to act safely.

The distinction between receiving information and being able to use it is particularly important. Parents had to manage medication, recognise clinically relevant changes, prioritise competing demands, and make decisions under pressure. Evidence from paediatric and family caregiving contexts similarly indicates that effective home-care capacity depends on the integration of knowledge, practical experience, confidence, and contextual support [38,39,40,41]. The present findings extend this understanding by showing that parents actively created systems to support care performance, including medication schedules, written records, stable routines, and task-sharing. These strategies reduced cognitive load and converted multiple, potentially fragmented demands into a more manageable pattern of care.

This process can be interpreted through Orem’s supportive–educative nursing system, in which nursing intervention is required when individuals or families can learn to meet care demands but need support, guidance, or education to do so safely [42]. In childhood cancer, however, care capacity is relational: it is exercised by parents on behalf of the child and shaped by the resources of the wider family system. Competence should therefore be assessed as a developing family capability rather than as an individual attribute or fixed prerequisite for discharge [43,44].

These findings support nurse-led discharge education that is practical, iterative, and responsive to parental performance [19]. Nurses can use demonstration, supervised practice, teach-back, and written or digital tools to assess whether parents can apply information rather than merely recall it. Such assessment should also consider emotional overload, health literacy, previous caregiving experience, and the availability of another caregiver, as these conditions may influence how safely complex responsibilities can be incorporated into daily life.

Discharge readiness should therefore be assessed not only by the information provided, but also by parents’ demonstrated ability to organise care, recognise warning signs, make decisions, and access professional support when uncertainty arises.

Parental competence should consequently be understood not as the endpoint of education, but as an evolving outcome of supported participation. Nursing care has a central role in calibrating responsibilities, identifying areas of difficulty, and reinforcing parents’ capacity to make informed decisions as they integrate complex care into family life.

### 4.3. Balancing Clinical Protection and Family Sustainability 

The transition home affected the family beyond the direct provision of care, requiring relationships, roles, and everyday routines to be reorganised around the child’s vulnerability. From a family-systems perspective, these changes are integral to the illness experience: demands affecting one member reverberate throughout the family, while patterns of family functioning shape how those demands are managed [34,35]. This phase therefore required families to reconcile the need to protect the child with the continuity of family life.

Returning home enabled proximity and reconnection but did not restore pre-illness normality. Contact with relatives, interactions between children, parental availability, and participation in ordinary activities remained conditioned by treatment requirements and perceived risk. This reflects the tension between continuity and change described in systemic models of family adaptation, whereby families seek to preserve relationships and routines that sustain identity while accommodating the demands of illness [34,36]. The present findings locate this negotiation in the early period at home, when the wish to resume family life remained inseparable from the perceived need for protection.

Siblings were particularly affected by this tension. Restrictions on proximity and shared activities, together with the concentration of parental attention on the child receiving treatment, altered their position within family life. Qualitative studies show that siblings of children with cancer may experience uncertainty, sudden separations, reduced involvement, and changes in the sibling relationship during transitions between hospital and home [14]. These challenges extend across family, hospital, school, and social contexts, underscoring that childhood cancer affects siblings as participants in the illness trajectory rather than as peripheral observers [15]. The present findings suggest that protecting one child may unintentionally constrain closeness and participation for others.

The feasibility of these arrangements also depended on the family’s practical and socioeconomic circumstances. Parents of children with cancer frequently adjust working hours, take unpaid leave, or withdraw from employment, with consequences that may persist beyond active treatment [8]. Distance from specialist paediatric oncology services may add travel demands, financial strain, and disruption to family routines [45]. Such conditions help explain why comparable clinical recommendations may generate different levels of burden across families.

These findings reinforce that adaptation cannot be attributed to parental competence alone. Rather, it emerges from the interaction between caregiving demands, family relationships, employment, social support, and access to specialist care [25,26,28]. Where resources are limited, responsibilities may become concentrated in one caregiver, increasing exhaustion and reducing the family’s capacity to preserve relationships and ordinary activities.

A clinically appropriate care plan may therefore remain difficult to sustain within a family’s relational, occupational, geographical, or material circumstances. Family-centred care requires assessment of the family as the unit of care, including the distribution of responsibilities, sibling needs, employment constraints, available support, and proximity to specialist services [16,17,18]. This assessment can identify where recommendations require adaptation or where additional professional and social support is needed.

Family sustainability should not be equated with returning to previous normality. Rather, it involves establishing a workable form of everyday life in which care demands, necessary precautions, and meaningful family relationships can coexist. Nursing support can contribute by anticipating the wider effects of care recommendations, including siblings in age-appropriate preparation, and mobilising resources that reduce the risk of isolation and caregiver exhaustion.

### 4.4. Limitations

These findings should be considered in light of the study context and design. The present analysis focuses on a major analytical category that emerged within a broader grounded theory study rather than from a study designed exclusively around hospital discharge from the outset. Participants were interviewed at different points in the childhood cancer trajectory, with the interval between the analysed discharge and the interview ranging from approximately 8 months to 7 years. Accounts were therefore retrospective to varying degrees and may have been shaped by subsequent treatment experiences, complications, recovery, or bereavement. Clinical heterogeneity contributed to conceptual variation but may limit transferability to more homogeneous clinical groups. Two participants from the broader study were not represented in the focused analysis because their joint interview did not contain substantive material concerning the analysed discharge transition.

Joint interviewing in some cases, prior professional familiarity between the lead researcher and some participants, and translation of quotations from Portuguese into English may have influenced how experiences were articulated or represented, despite the reflexive procedures and safeguards described above. The study was conducted in a single specialised paediatric oncology setting in Portugal. The study period also partly overlapped with the later COVID-19 era, and broader infection-prevention norms may therefore have influenced some practices concerning masks, distancing, visitors, or social contact. In addition, the analytically specific post-HSCT transition involved higher-intensity caregiving and infection-prevention requirements, which may limit the transferability of some practices to other treatment contexts. Finally, the analysis reflects parental accounts and does not directly represent the perspectives of children, siblings, or healthcare professionals.

Future research should evaluate how tailored discharge preparation and structured post-discharge support influence parental readiness, caregiving confidence, and the sustainability of care at home.

## 5. Conclusions

The first hospital discharge in childhood cancer represents more than a change in care setting. It marks a transfer of responsibility in which parents must establish safety at home while assuming increasingly complex caregiving responsibilities and reorganising family life around the child’s clinical vulnerability.

The findings show that safety is actively constructed through vigilance, preventive practices, environmental adaptation, caregiving organisation, and the mobilisation of professional and informal support. This process develops progressively as parents gain experience and confidence but is shaped by the child’s clinical condition and by the family resources and circumstances within which care must be sustained.

Nursing support should therefore extend beyond technical discharge instruction to include progressive preparation, assessment of parental readiness, practical support for the development of caregiving competence, accessible nursing guidance and coordinated multidisciplinary support after discharge, and attention to the wider family conditions that influence the sustainability of care at home.

## Figures and Tables

**Figure 1:**
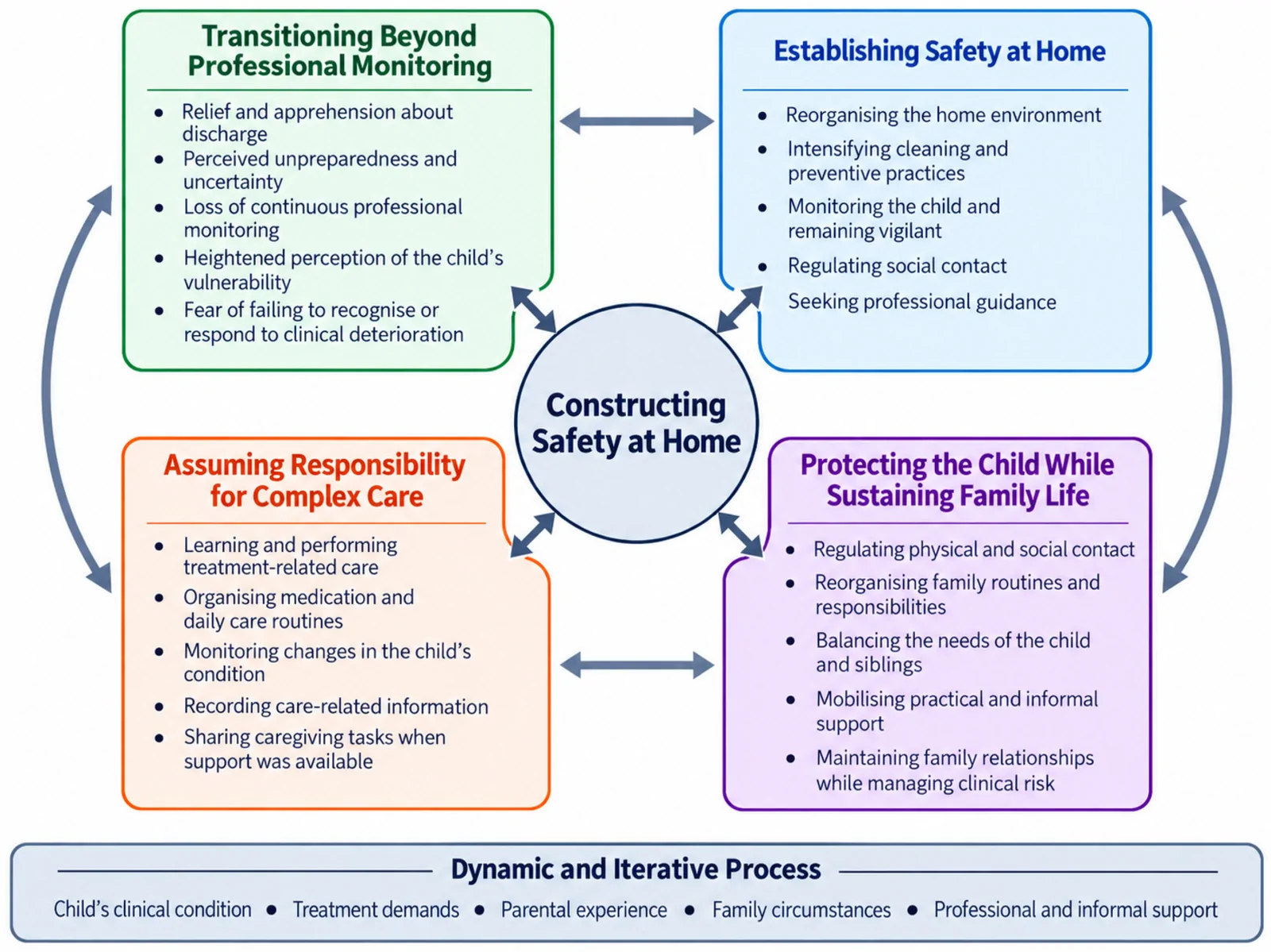
Constructing safety at home as a dynamic and iterative process following the child’s first hospital discharge during cancer treatment.

**Table 1:** Characteristics of participants, interviews, and children.

Characteristic	Category	n
Parent characteristics (n = 14)		
Age	30–39 years	4
	40–49 years	7
	50–59 years	3
Relationship to the child	Mother	9
	Father	5
Marital status	Married/cohabiting	13
	Divorced	1
Number of children	1	5
	2	7
	3	2
Employment status	Employed	11
	Unemployed or in precarious employment	3
Leave of absence from work to care for the child *	Yes	7
	No	4
Educational level	Upper secondary education	2
	Higher education	12
Interview characteristics (n = 13 interviews)		
Interview format	Individual	12
	Joint parental interview	1
Child and illness-trajectory characteristics (n = 10 children)		
Age at diagnosis	<1 year	1
	2–5 years	5
	6–12 years	2
	13–17 years	2
Diagnosis	Haematological malignancy	8
	Solid tumour	2
Illness trajectory at interview	Active treatment	3
	Completed curative treatment	5
	Child had died	2
Haematopoietic stem cell transplantation (HSCT) before interview	Yes	3
	No	7
Approximate interval between analysed discharge and interview	<1 year	3
	1–5 years	6
	>5 years	1

* Among the 11 participants who were employed.

## Data Availability

The qualitative data generated and analysed during this study are not publicly available because they contain sensitive information that could compromise participant and institutional confidentiality. Access is therefore restricted in accordance with the ethical and confidentiality requirements of the approved study protocol.

## Supplementary Materials

The following supporting information can be downloaded at: https://www.mdpi.com/article/10.3390/nursrep16100348/s1, Supplementary File S1: COREQ (COnsolidated criteria for REporting Qualitative research) Checklist; Supplementary File S2: Open Interview Guide.

## Public Involvement Statement

No public involvement in any aspect of this research.

## Guidelines and Standards Statement

This manuscript was drafted in accordance with the Consolidated Criteria for Reporting Qualitative Research (COREQ) guidelines for qualitative research [21].
